# Perspectives on Exertional Rhabdomyolysis

**DOI:** 10.1007/s40279-017-0689-z

**Published:** 2017-03-22

**Authors:** Eric S. Rawson, Priscilla M. Clarkson, Mark A. Tarnopolsky

**Affiliations:** 1 Department of Health, Nutrition and Exercise Science, Messiah College, One College Avenue Suite 4501, Mechanicsburg, PA 17055 USA; 2 Department of Kinesiology, University of Massachusetts, Amherst, MA USA; 3 Department of Pediatrics and Medicine, McMaster University, Hamilton, ON Canada

## Abstract

Exertional (exercise-induced) rhabdomyolysis is a potentially life threatening condition that has been the subject of research, intense discussion, and media attention. The causes of rhabdomyolysis are numerous and can include direct muscle injury, unaccustomed exercise, ischemia, extreme temperatures, electrolyte abnormalities, endocrinologic conditions, genetic disorders, autoimmune disorders, infections, drugs, toxins, and venoms. The objective of this article is to review the literature on exertional rhabdomyolysis, identify precipitating factors, and examine the role of the dietary supplement creatine monohydrate. PubMed and SPORTDiscus databases were searched using the terms rhabdomyolysis, muscle damage, creatine, creatine supplementation, creatine monohydrate, and phosphocreatine. Additionally, the references of papers identified through this search were examined for relevant studies. A meta-analysis was not performed. Although the prevalence of rhabdomyolysis is low, instances still occur where exercise is improperly prescribed or used as punishment, or incomplete medical history is taken, and exertional rhabdomyolysis occurs. Creatine monohydrate does not appear to be a precipitating factor for exertional rhabdomyolysis. Healthcare professionals should be able to recognize the basic signs of exertional rhabdomyolysis so prompt treatment can be administered. For the risk of rhabdomyolysis to remain low, exercise testing and prescription must be properly conducted based on professional standards.

## Introduction

Exertional rhabdomyolysis continues to be reported in healthy and patient populations despite efforts from the medical and research communities to provide guidelines about this potentially life-threatening condition. As an example, in 2006, exertional rhabdomyolysis was reported in a 12-year-old student who was forced to do >250 squat jumps as punishment for talking in class [1]. While such troubling cases appear in the literature, the reports of group “outbreaks” of rhabdomyolysis are even more disconcerting. More than half of the McMinnville, Oregon, USA, high school football players who entered immersion camp in August of 2010 went to the hospital with rhabdomyolysis following intense unaccustomed exercise [2, 3]; 12 were admitted and three had fasciotomies to treat compartment syndrome of the upper arm. Similarly, 12 University of Iowa football players were hospitalized with rhabdomyolysis following intense exercise in January 2010 [4]. Historically, there have been other “outbreaks” of rhabdomyolysis [5] and exertional (exercise-induced) rhabdomyolysis [6, 7].

While many researchers/clinicians recognized exertional rhabdomyolysis, numerous theories emerged from the media and hundreds of comments were posted on the internet; many appeared determined to find a cause of the “outbreak” besides over-exertion/unaccustomed exercise. Although many factors can influence the likelihood of rhabdomyolysis, the dietary supplement creatine appeared to be a popular focus of these theories and comments despite a body of literature that demonstrates little or no association between creatine supplements and rhabdomyolysis [8, 9]. Consequently, the purpose of this article is to (1) review the literature on exertional rhabdomyolysis and identify precipitating factors and (2) review the effects of creatine supplementation on markers of muscle damage and exertional rhabdomyolysis. PubMed and SPORTDiscus databases were searched using the terms rhabdomyolysis, muscle damage, creatine, creatine supplementation, creatine monohydrate, and phosphocreatine. Additionally, the references of papers identified through this search were examined for relevant studies. All articles in which individuals were ingesting creatine supplements were included. A meta-analysis was not performed.

## Rhabdomyolysis

### Background

Rhabdomyolysis by definition means damage to skeletal muscle. It was initially called “crush syndrome,” as it was identified in 1940 during the London Blitz in patients whose limbs were crushed. These patients survived their initial injury, only to die of renal failure several days later. The causes of rhabdomyolysis are numerous and can include direct muscle injury (e.g., crush, bites, electrical injuries, repetitive blows), unaccustomed exercise (especially with eccentric/muscle-lengthening contractions), ischemia (e.g., tourniquets, immobilization, compartment syndrome), extreme temperatures, electrolyte abnormalities (e.g., hypokalemia, hyponatremia), endocrinologic conditions (e.g., diabetic ketoacidosis, hypothyroidism), genetic disorders (e.g., metabolic myopathies, muscular dystrophy, pseudometabolic myopathy), autoimmune disorders (e.g., polymyositis, overlap myositis), infections (e.g., *Staphylococcus aureus* sepsis, viral myositis, necrotizing fasciitis), drugs (e.g., statins), toxins (e.g., hemlock, toxic mushrooms), and venoms (e.g., bites, stings) [10]. Regardless of the cause, damage to the muscle results in increased intracellular calcium, which in turn activates proteases and causes necrosis. As the focus of this review is exertional rhabdomyolysis, the reader is directed elsewhere for a complete description of the etiology and non-exercise causes of rhabdomyolysis [10, 11].

Delayed-onset muscle soreness (DOMS) experienced a day or so after strenuous unaccustomed exercise is an indicator of muscle damage and signifies a very mild form of rhabdomyolysis. This becomes clinically relevant when there is extensive damage to the muscle fibers, the pain is severe, and large amounts of muscle proteins are released into the blood stream (e.g., creatine kinase [CK], lactate dehydrogenase [LDH], and myoglobin). While other proteins are cleared by the reticulo–endothelial system, myoglobin is cleared by the kidneys. Myoglobin, and likely other proteins, can precipitate in the kidney or be directly nephrotoxic (oxidative stress), resulting in acute renal failure that—if left untreated—could be life threatening. A cardinal sign that an individual should seek immediate treatment is dark-colored urine (pigmenturia [brown, red, tea, or cola colored]); this occurs because the kidneys are clearing myoglobin from the blood and it spills over into the urine. However, myoglobinuria without rhabdomyolysis has been described in ultra-marathon runners [12], which further complicates diagnosis and treatment.

It can be difficult to know when an individual crosses the line from a normal physiological response to exercise to clinically relevant rhabdomyolysis. Blood CK levels increase in the hours following muscle damage and remain elevated for several days. CK is easily assessed in the serum and is a sensitive, but not specific, marker of muscle tissue damage. While some would argue that CK values >15,000 IU/l are predictive of renal impairment, this is likely population specific. For instance, we showed post-exercise CK > 2000 IU/l (*n* = 111) and >10,000 IU/l (*n* = 51) in volunteers who performed 50 maximal eccentric contractions of the elbow flexors. Despite the markedly increased CK of these research volunteers, none experienced visible myoglobinuria or required treatment for impaired renal function [13]. Kenney et al. [14] examined data from military recruits in basic training (*n* = 499) and, although none developed clinical exertional rhabdomyolysis, mean CK values were 734, 1226, and 667 IU/l on days 3, 7, and 14 post-exercise (normal adult values: males 52–336 IU/l; females 38–176 IU/l). The highest CK reported was 35,056 IU/l. The authors suggested that because CK levels consistently exceeded normal values, >50 times the traditional upper limit would be more specific for this population. Thus, exertional muscle damage produced by eccentric exercise in healthy individuals can cause profound CK and myoglobin elevations without renal impairment, and elevated CK is likely a population-specific bio-marker.

Treatment involves intravenous fluids with or without bicarbonate to alkalinize the urine for 3–7 days, with the goal to dilute myoglobin and other potential nephrotoxic substances. The damage to muscle may also produce myoedema, leading to compartment syndrome where the connective tissue surrounding the muscle restricts the swelling, increases the pressure, and limits blood flow to the muscle, causing a “vicious cycle” of tissue necrosis. Treatment of acute compartment syndrome involves cutting the connective tissue (fasciotomy) to allow the muscle to swell without restriction, thus maintaining blood flow. When rhabdomyolysis involves the development of acute renal failure or compartment syndrome, it becomes clinically relevant [15]. Of interest to many clinicians and practitioners is the issue of “return to play/duty/physical activity.” A full discussion is beyond the scope of this paper. Although there are no standard guidelines, we refer the reader to the excellent review by O’Connor et al. [16], who suggest that three issues must be addressed: (1) recurrence risk, (2) timeline of safe return, and (3) potential restrictions.

Exertional or exercise-induced rhabdomyolysis has been reported in case studies following excessive unaccustomed exercise in several different scenarios including, military training [17–27], physical education class [6], mountaineering [28], supervised [29–32] and unsupervised [33–37] strenuous training, and marathon running [12, 38–41]. Several factors can increase the risk of exertional rhabdomyolysis, including an increase in exercise intensity/duration or using different muscle groups (unaccustomed strenuous exercise), muscle contraction type (eccentric/lengthening > concentric), infection, medication use, dietary factors (e.g., hydration), environmental factors (extremes of heat and cold), sex (males > females), and genetic factors (reviewed by Huerta-Alardin et al. [11]). Potential precipitating and mitigating factors in exertional rhabdomyolysis are discussed in the following sections.

### Resistance vs. Endurance Exercise

Performance of unaccustomed exercise is the primary determinant of exertional rhabdomyolysis. Most often this occurs in trained individuals who dramatically increase their training workout, train a different muscle group, or use a different contraction type, sometimes when encouraged and motivated to overexert themselves by a coach or personal trainer [32]. In untrained individuals, this can occur during the start of an exercise training program, especially when a personal trainer or coach encourages overexertion [32].

The muscle contractions that cause the most muscle damage are those where the muscle lengthens as it is attempting to contract (also referred to as eccentric in classical literature), for example lowering a weight during a biceps curl. In the gym, these contractions are referred to as “negatives.” High-force (high-resistance) lengthening contractions place strain on the muscle fibers and cause damage to protein structure, releasing muscle proteins into the circulation [42]. Some aerobic exercises can be biased toward lengthening contractions, such as downhill running [43]. In the laboratory, we use such contractions to study mild forms of rhabdomyolysis (not clinically relevant) [13, 44–46]. We have found that performance of laboratory eccentric exercise results in DOMS/pain, prolonged losses in strength and range of motion, and release of muscle proteins into the circulation [47].

Endurance (aerobic) exercise typically does not result in clinically relevant rhabdomyolysis. While rare instances of rhabdomyolysis occur in marathon runners [40], the degree to which renal failure is a consequence of rhabdomyolysis or occurs independent of rhabdomyolysis (i.e., severe dehydration, hyperhydration, or hyponatremia) is not known. McCullough et al. [48] examined kidney function in 25 marathon runners 4 weeks before, immediately after, and 24 h after a race. Of the runners, 40% showed evidence of acute kidney injury (based on increase in serum creatinine) immediately following the race, which resolved in the next 24 h. Data from the Comrades Marathon in South Africa showed that in an 18-year period (1969–1986), only 19 cases of acute renal failure were reported, whereas the total number of participants ranged from 2000 to 10,000 per year [49]. In cases of marathon running, it appears that renal failure can occur without a profound increase in muscle proteins in the circulation, likely due to varying combinations of dehydration, superimposed illness, hyperthermia, and drugs leading to lowered renal perfusion [49]. For example, MacSearraigh et al. [50] reported nine cases of acute renal failure in the Comrades Marathon; several runners had taken analgesics and four may have had a viral or bacterial infection. Another report from the Comrades Marathon [49] found four cases of acute renal failure; two had an infection prior to the race and all four ingested analgesics prior to the race. Analgesics, particularly non-steroidal anti-inflammatory drugs (NSAIDs), can reduce renal perfusion, leading to a depressed glomerular filtration rate [51]. Furthermore, one case report described a marathon runner who had taken NSAIDs before the race for a viral infection and who collapsed at the finish line in cardiac arrest; he was later diagnosed with a muscle myopathy [52]. Several factors may together create a situation that increases the risk for acute renal failure in marathon runners, including pre-existing viral or bacterial infection and/or use of analgesics and NSAIDs.

### Effects of Hydration

Clinically relevant rhabdomyolysis is often associated with unaccustomed, strenuous exercise in a hot environment, especially where dehydration is a factor [15]. Reduced hydration impairs the ability of the kidneys to clear myoglobin and other potential myotoxic substances, consequently increasing the risk of myoglobin precipitation, renal cast formation, and possibly local factors intrinsic to the kidney such as oxidative stress (ischemia–reperfusion), increasing the risk for renal failure. Although a risk factor, dehydration does not have to be present for clinically relevant rhabdomyolysis to manifest [31, 32]. Somewhat paradoxically, rhabdomyolysis can also occur in the presence of hyperhydration during exercise, wherein associated hyponatremia is a likely etiological factor. Of 400 starters of the 2009 Western States 161-km Endurance Run, five presented with rhabdomyolysis and hyponatremia [53]. Although this small number of cases seems inconsequential, clinicians must be aware that the treatments for rhabdomyolysis (intravenous isotonic fluid) and hyponatremia (water restriction or intravenous concentrated/hypertonic fluid) are incongruous. The relationship, causal or independent, between hyponatremia and rhabdomyolysis is currently unknown. It is unclear how over-hydration leads to a higher risk of rhabdomyolysis, but hyponatremia is clearly a key factor.

### Sex Differences

Rhabdomyolysis occurs more frequently in men than women. Alpers and Jones [54] reported that, of 63 cases of exertional rhabdomyolysis in military trainees, 88.9% were men, although the overall population of trainees without rhabdomyolysis was 75.5% men. Using our laboratory exercise regimen to induce a mild form of rhabdomyolysis, we found that a higher percentage of women than of men experienced profound (>70%) loss in strength immediately after high-force eccentric exercise [55]. However, men showed greater average increases in CK, and more men than women were high CK responders [55]. Reportedly, men usually show a greater immune response to eccentric contractions [56]. Although there is currently no explanation for these sex differences, some models indicate a protective role of estrogen [44, 56].

### Children and Adolescents

Rhabdomyolysis in children is rare. One published case describes a 17-year-old boy who presented with myalgia, weakness, and dark urine 6 days following the start of high-school football practice [57]. Myalgia was first noted on the evening after the first practice, but the boy continued the practice sessions for the next 5 days. Serum CK was 96,000 U/l, and urine dipstick was positive for the presence of blood. Lin et al. [6] presented a brief report of 119 high-school students aged 17–18 years who performed 120 push-ups in 5 min as part of a physical education class. The post-exercise serum CK values ranged from 55 to 174,260 U/l. Many of the students developed muscle pain and dark urine 2–4 days after the exercise. Most were treated with oral hydration, but 20 students were hospitalized. We reported a case of an 18-year-old football player following a supervised practice session led by the team’s strength and conditioning coach [31]. The day after this session, the player experienced extreme pain and dark urine and sought treatment at a local emergency department. Hospitalization resulted in a diagnosis of rhabdomyolysis based on myoglobinuria, muscle pain, and extremely elevated circulating CK values (>130,000 U/l). Following 8 days of hospitalization with intravenous fluids, the patient recovered without complications. The strength coaches, athletic trainer, and team physician missed the classic signs of rhabdomyolysis (extreme pain and brown urine) and did not recommend that the player get treatment. We also reported [29] exertional rhabdomyolysis in a 12-year-old boy who participated in an indoor physical education class where excessive (between 250 and 500) repetitive squat jumps were performed as punishment for talking in class. The boy, who reported intense muscle soreness in the thighs and dark urine 2 days post-exercise, was brought to the emergency room by his parents. His serum CK was 92,115 U/l, and urinalysis indicated the presence of blood and protein. He was transferred to another hospital that evening, admitted, and treated for 7 days. His serum CK rose to 244,006 U/l at 4 days post-exercise. The boy was discharged after 7 days of hospitalization, at which time CK was 9101 U/l. The other children in the class were not hospitalized.

### Other Precipitating Factors

Many of the case studies cited above illustrate that only a small percentage of individuals exposed to the same exercise conditions experience rhabdomyolysis. For example, one report stated there were 22.2 cases of exertional rhabdomyolysis per 100,000 military trainees per year [54]. Another case in point is the Iowa football team mentioned in Sect. 1. Only 13 of about 100 players who trained that day were hospitalized and treated for clinically relevant rhabdomyolysis. In our laboratory, where we induce a safe form of rhabdomyolysis, a small percentage of subjects are high responders [58]. These high responders experience severe muscle pain, profound swelling, extreme muscle weakness, and/or large elevations of CK in the blood. There are known genetic factors that will predispose a person to rhabdomyolysis, such as sickle cell trait [59–61], carnitine palmitoyltransferase (CPT) deficiency, and McArdle disease [15]. Although a genetic disorder may explain episodes of recurrent rhabdomyolysis after minimal to moderate exertion, they are not typically associated with the first bout of exertional rhabdomyolysis after strenuous unaccustomed exercise. Given the huge variance in CK elevation and muscle damage seen in response to controlled eccentric exercise performed in the laboratory setting [42, 46, 47, 56, 62, 63], it is likely that polygenetic factors lead to inter-individual rhabdomyolysis risk as opposed to monogenetic disorders such as fatty acid oxidation defects (e.g., CPT2 deficiency) or glycogen storage diseases (e.g., McArdle disease). We [64, 65] and others [66–68] have found that certain single nucleotide polymorphisms (SNPs) may be associated with a high response. SNPs are variations in a single nucleotide in the DNA. There are thousands of these variations, and typically SNPs exert an effect in coordination with other SNPs. Thus, it is likely that many SNPs may contribute to making an individual more susceptible to exertional muscle damage (polygenetic risk variance). Although most cases of exertional rhabdomyolysis are likely due to polygenetic risk and overexertion (often under external influence to push beyond the intrinsic signals telling one to stop—i.e., not “listening to the body”), it is important to identify the small fraction of patients who present with rhabdomyolysis who have an underlying monogenetic metabolic myopathy.

## Metabolic Myopathies and Rhabdomyolysis

### Overview of Metabolic Myopathies

Metabolic myopathies are genetically determined inborn errors of metabolism that impair the bioenergetic pathways responsible for fat, amino acid, and mitochondrial or carbohydrate oxidation. Under circumstances of cellular metabolic stress, these disorders can result in an energy crisis (low energy charge) in the cell that can lead to rhabdomyolysis. It is important to note that metabolic myopathies are a rare cause of exertional rhabdomyolysis but are nevertheless important to recognize since important preventive and acute treatment strategies exist as does genetic counseling for patients and their families.

In addition to the inborn errors of metabolism, several of the muscular dystrophies can present with a “pseudometabolic” pattern where physical activity can trigger exertional and post-exertional myalgias with rhabdomyolysis, hyperCKemia and eventually myoglobinuria [69]. One clue to an underlying genetic muscular dystrophy is muscle weakness and hyperCKemia that remain even after resolution of the rhabdomyolysis event, whereas the inborn errors of metabolism usually result in full recovery of strength and normalization of hyperCKemia, with the exception of McArdle disease where hyperCKemia is a chronic feature.

### Fatty Acid Oxidation Defects

The most common fatty oxidation defects are related to mutations in CPT2, very long chain acyl-CoA dehydrogenase (VLCAD), and trifunctional protein (TFP) proteins. These patients can present with a rhabdomyolysis phenotype (e.g., myalgia, pigmenturia) precipitated by longer-term exercise, fasting, or concomitant illness. The diagnosis is best investigated by acyl-carnitine profiling in blood or fibroblast culture; however, in some of the milder cases the defect can only be seen only during a metabolic crisis or with superimposed stress (fasting or exercise). During an acute bout of rhabdomyolysis in β-oxidation defects, free fatty acid levels in the serum are often high and ketones are low (β-OH butyrate). Acyl-carnitine profiling in serum can often be used to target the most likely genetic mutational analysis, and, once a specific defect is suspected, a targeted mutational analysis of the gene in question can be performed (reviewed by van Adel and Tarnopolsky [70]).

The best treatment for these disorders is to avoid precipitating factors such as exercising in the fasted state or with a superimposed infection. A high-carbohydrate diet is beneficial [71], and although carbohydrate supplementation immediately prior to and during exercise is logical and anecdotally of significant benefit to patients, one study suggested that it was of limited benefit compared with intravenous glucose [72]. Nevertheless, given the absence of side effects and the conceptual basis behind the strategy, carbohydrate consumption during exercise is worth evaluating on a patient-by-patient basis. Supplementation with medium-chain triglyceride oil is not likely to be of any significant benefit and although riboflavin has not been definitely shown to be of benefit, it is essentially non-toxic and some patients may consider a trial of 100 mg twice daily. Another strategy, using the anapleurotic substance triheptanoin, may be of some benefit, although availability of this substance is a practical challenge; clinical studies are underway. More recently, there is some evidence that a peroxisome proliferator-agonist receptor (PPAR) agonist called bezafibrate (fibric acid derivative used for hypertriglyceridemia) can enhance beta oxidation in vitro [73] but likely only in ~25% of cases [74], and studies are underway to see whether this helps patients with fatty acid oxidation defects. Certainly, if a patient with a fat oxidation defect had hypertriglyceridemia requiring medical therapy, a fibrate would be a drug of first choice.

### Disorders of Carbohydrate Metabolism/Glycogen Storage Disease

Patients with defects in glycolysis or glycogenolysis usually experience muscle cramps with higher-intensity activity, occasionally followed by rhabdomyolysis and pigmenturia. The cramps are often quite painful and usually resolve with rest for several minutes. In the glycogenolytic defects (but not the glycolytic defects), there is a “second wind phenomenon” whereby the exercise feels easier after a period of reduced-intensity exercise; this is due to the delivery of blood-born substrates. Occasionally, swelling can ensue within the muscle and lead to compartment syndrome. The most common glycogen storage disease affecting muscle is McArdle disease, which is due to a genetic defect in myo-phosphorylase, and several hundred mutations have been documented. Rare cases of phosphorylase b kinase deficiency have been documented, but the clinical phenotype is usually milder. The second most common defect is a deficiency of phosphofructokinase (PFK), which causes a similar clinical picture to that of McArdle disease and may also be associated with hemolysis and does not show the second wind phenomenon (for a review, see van Adel and Tarnopolsky [70]).

Patients with glycogenolytic and glycolytic defects will have no (or very attenuated) increase in lactate in response to a semi-ischemic or non-ischemic forearm exercise and will show enhanced ammonia rise [75]. The increase in ammonia is due to an increased reliance on the adenylate kinase and the adenosine monophosphate (AMP)–deaminase pathway as a cellular attempt to provide an alternative source of anaerobic adenosine triphosphate (ATP). A normal forearm exercise test rules out essentially all of the glycolytic and glycogenolytic defects except for phosphorylase b kinase deficiency, where the blunting of lactate rise is best seen with aerobic exercise [76]. A muscle biopsy can be checked for specific phosphorylase or PFK enzyme activity at the histochemical level. Periodic acid–Schiff (PAS) staining usually shows an increase in glycogen, and electron microscopy shows a non-specific increase in non-membrane-bound glycogen in the glycolytic and glycogenolytic defects. Although CK increases acutely with rhabdomyolysis, most patients with McArdle disease will have chronically elevated CK activity in blood and some will have myogenic hyperuricemia and develop gout (due to the flux through the adenylate kinase → AMP deaminase → xanthine oxidase pathway).

Paradoxically, the most effective treatment for McArdle disease is consistent exercise training [77, 78], and exercise tolerance is clearly enhanced with pre-exercise carbohydrate ingestion [79]. Exercise needs to be undertaken in the fed state except in PFK deficiency, where exercise should be completed in the fasted state because carbohydrate feeding attenuates lipolysis and makes PFK-deficient patients worse [80]. Carefully supervised and gradually progressive exercise significantly improves exercise capacity in patients with McArdle disease [78], and we also have seen very dramatic results with carefully planned and gradually progressive exercise in our patients with McArdle disease. One study showed that low-dose creatine monohydrate enhanced power output in patients with McArdle disease [81]. High-dose creatine supplementation actually impaired exercise capacity in patients with McArdle disease [82], possibly through an inhibition of PFK at the higher creatine dose levels. Several studies have clearly shown that pre-exercise carbohydrate supplementation can enhance exercise energy output because the plasma glucose entering the cell can be immediately oxidized for energy since it does not need to first form glycogen before glycolytic flux. Other strategies such as the higher protein diet and pyridoxine supplementation have been evaluated and have not been particularly effective in clinical trials but could be considered in isolated cases [83].

### Mitochondrial Myopathies

The mitochondrial myopathies are those defects due to primary genetic defects affecting components of the electron transport chain (ETC). Given that mitochondrial diseases lead to aerobic energy deficits (low maximal oxygen consumption [*V*O_2max_]), most patients have moderate to severe exercise intolerance. Although rhabdomyolysis as a primary manifestation of a mitochondrial myopathy is rare, those with rhabdomyolysis present under metabolic conditions similar to those in patients with fatty acid oxidation defects (fasting, prolonged exercise). Many mitochondrial DNA (mtDNA) and nuclear DNA (nDNA) mutations cause mitochondrial cytopathies, but only a few have been associated with exercise-induced rhabdomyolysis. The most common mutations that lead to rhabdomyolysis/myoglobinuria are the cytochrome *b* mutations [84]; however, rhabdomyolysis/myoglobinuria can be seen in patients with mitochondrial myopathy due to mutations in other mtDNA genes, including cytochrome c oxidase subunits [85], multiple mtDNA deletions [86], leu-transfer RNA (tRNA) (3243 A>G; 3260 A>G) [87], and ile-tRNA [88], and a mutation has been found in a mitochondrial iron-sulfur cluster assembly (ISCU) protein [89].

The workup of patients with mitochondrial cytopathy is complex and beyond the scope of this review; however, readers are directed towards several review papers for a more in-depth discussion [70, 90]. In brief, plasma lactate is elevated in approximately 65% of patients with mitochondrial cytopathies; however, care must be taken to ensure that the sample is collected and transported on ice to avoid false positives. Plasma amino acid analysis may show an elevation of alanine. Urine organic acid analysis may show elevations of lactate, pyruvate, malate, or 3-methyl glutaconic acid. Definitive testing requires a muscle biopsy with light and electron microscopic evaluation. Common light microscopic abnormalities include ragged red fibers and cytochrome *c* oxidase negative fibers. Electron microscopic analysis often shows pleomorphism and increased number of mitochondria that may contain paracrystalline inclusions (crystallized mitochondrial CK). Enzymatic activity of the ETC enzymes often helps identify a specific mutation in one of the complexes or multiple defects sometimes seen in the tRNA or ribosomal RNA defects. Genetic analysis for specific point mutations is best done on muscle-derived DNA; mtDNA deletions are rarely, if ever, seen in white blood cell-derived DNA samples and ideally should be run from muscle biopsy-derived mtDNA. Although mutations in mtDNA are well described [70], an increasing number of mutations have been identified in nDNA, and the discovery of the ISCU mutation [88, 89] represents a nuclear gene defect associated with rhabdomyolysis.

The treatment of mitochondrial myopathies is complex and evolving, but most patients are placed on a “mitochondrial cocktail” of co-factors, antioxidants, and alternative energy sources (i.e., creatine monohydrate) [91, 92]. We also measure plasma carnitine levels and replace deficiencies to avoid secondary fatty acid deficiency-like triggering of rhabdomyolysis during exercise. Strategies such as avoiding unaccustomed exercise during fasting or concurrent illness are also recommended. Perhaps the most effective therapy is carefully titrated endurance and resistance exercise training to raise the threshold for triggering symptoms of weakness, muscle fatigue, and rhabdomyolysis [93].

### Statin Myopathies

Statins are effective lipid-lowering drugs that block the enzyme 3-hydroxy-3 methylglutaryl co-enzyme A (HMG-CoA) reductase. The statins are a very safe group of medications as a whole, but myalgias can be seen in up to 5% of all patients taking the medication, and ~0.1% of all users will experience rhabdomyolysis, defined as a CK elevation to >10 times the upper limit of normal [94].

The cause of statin myopathies is unclear, and many theories have been put forth, including reduced coenzyme Q10 synthesis, lower cholesterol/altered sarcolemmal fluidity, reduced phrenylation of structural proteins, and activation of apoptosis. Genetic predispositions for the statin myopathies such as the metabolic myopathies [95] and polymorphisms in LPIN 1 [96] are likely. Acute exercise-induced hyperCKemia appears to be enhanced in patients receiving statins, and this is true for both endurance- and resistance-type exercise and can even occur in well-trained athletes [94]. Fortunately, some people can avoid the use of statins and other hypercholesterolemic medications with exercise training and dietary interventions and obviate the potential interaction between statins and exercise by eliminating the need for the statin.

## Creatine Supplementation and Exertional Rhabdomyolysis

### Creatine Metabolism and Supplementation

Creatine is a non-essential nutrient that is consumed in the diet and produced in the body from amino acids. Approximately 95% of the body’s creatine is stored in skeletal muscle as creatine or phosphocreatine, where it is involved in energy metabolism. Both creatine and phosphocreatine are non-enzymatically degraded to creatinine and excreted in the urine. High-intensity exercise tasks (e.g., 100-m sprint, one down in gridiron football, one set of squats) rely heavily on muscle phosphocreatine to resynthesize ATP to support muscle contraction. Skeletal muscle phosphocreatine and creatine content can be increased about 20% with oral creatine ingestion using either a brief high-dose protocol (about 20 g/day for 5 days) [97] or a longer-term lower-dose protocol (about 3 g/day for about 30 days) [98]. Improved exercise performance, particularly high-intensity exercise, following creatine supplementation, is a consistent finding in the literature (reviewed by Gualano et al. [8], Bemben and Lamont [99], Branch [100], and Rawson and Volek [101]) and occurs with both the high dose (reviewed by Gualano et al. [8]) and lower-dose [102, 103] supplementation protocols. Creatine exerts an ergogenic effect through multiple mechanisms involving pre-, during, and post-exercise enhancements. These include increased pre-exercise muscle creatine and phosphocreatine [97, 98] and glycogen [104–106] (reviewed by Volek and Rawson [107]), faster phosphocreatine re-synthesis during recovery periods [108, 109], reduced post-exercise inflammation/muscle damage [110–115], increased expression of growth factors [116–120], faster post-exercise strength recovery [112], lower protein degradation [121], and increased training volume [122] (reviewed by Rawson and Persky [123]). Additionally, creatine supplementation has been shown to improve muscle function in various patient populations (e.g., dystrophy, aging) (reviewed by Gualano et al. [8], Kley et al. [124], Rawson and Venezia [125], and Tarnopolsky [126]) and has an excellent safety profile (reviewed by Gualano et al. [8] and Persky and Rawson [9]).

### Creatine Supplementation, Muscle Damage, and Rhabdomyolysis

The first connection between creatine supplementation and rhabdomyolysis was likely made in 1997, when three National Collegiate Athletic Association (NCAA) wrestlers died within a 2-month period (official causes of death were unknown, hyperthermia, and rhabdomyolysis) [127]. Each had been using rapid weight loss techniques (e.g., intense exercise wearing vapor-restrictive clothing while in a sauna) to qualify for a lower weight class. This event was covered by major media outlets and forced the NCAA to reevaluate weigh-in procedures, weight class limits, and weight loss tactics. Creatine was at the center of the storm, although the Centers for Disease Control (CDC) and the US FDA did not implicate creatine. In fact, the often cited article published in *Morbidity and Mortality Weekly Reports* that described the wrestler’s weight loss tactics and subsequent deaths does not even mention the word creatine [127]. However, a myth was born, and the erroneous connection between creatine supplementation and rhabdomyolysis still exists today.

Oddly, few publicly questioned why athletes struggling to lose weight would be ingesting a dietary supplement known to increase body mass. Additionally, few pointed to ephedrine, a banned substance that is remarkably under-reported in NCAA athletes compared with non-NCAA athletes and a seemingly ideal supplement for wrestlers (but not condoned by the authors) as it decreases appetite, increases metabolic rate, and decreases body mass (reviewed by Rawson and Clarkson [128]). Ephedrine increases heart rate and blood pressure in young adults [129], has a higher absorption rate and peak plasma concentration when combined with dehydration [130], can be cardio-toxic [131], and was clearly being used by wrestlers at the time [132]. When the 2010 rhabdomyolysis “outbreaks” happened in Oregon and Iowa, and despite more than 10 years of research on creatine supplementation, muscle damage, and rhabdomyolysis, creatine was implicated again in spite of little to no evidence to support the claim.

### Creatine Supplementation and Resting Markers of Muscle Damage

Both Robinson et al. [133] and Mihic et al. [134] reported no effect of high-dose short-term (20 g/day for 5 days) and low-dose longer-term (3 g/day for 8 weeks) creatine supplementation on blood CK activity in physically unstressed subjects. Additionally, Robinson et al. [133] found no increase in serum CK activity in a group that performed 8 weeks of resistance training in addition to creatine ingestion (20 g/day for 5 days followed by 3 g/day for 8 weeks). Both groups reported no changes in renal function. Schilling et al. [135] and Kreider et al. [136] reported no differences in resting serum LDH and CK, respectively, in elite strength/power athletes. Following a high-dose creatine loading protocol, athletes in these studies ingested maintenance doses of creatine 5–10 g/day for 0.8–4 years [135, 136]. Collectively, these data indicate that creatine supplementation has no effect on bio-markers of muscle damage. The effects of creatine supplementation on markers of muscle damage at rest are summarized in Table 1 [133–136].

**Table 1:** Effects of creatine supplementation on markers of exercise-induced muscle-damage

Supplementation protocol and study design	Exercise stress	Biological outcomes	Functional outcomes	Perceived outcomes	Overall effect of creatine on muscle-damage	References
20 g/day for 5 days; DB, PC	Unstressed	No increase in resting CK	NA	NA	No effect	[134]
20 g/day for 1 day, 20 g/day for 5 days, followed by 3 g/day for 8 wks with training; DB, PC	Unstressed and supervised resistance exercise 3 × week	No increase in resting CK	NA	No reports of cramping or muscle injury	No effect	[133]
Mean loading dose 13.7 g/day, mean maintenance dose 9.7 g/day, duration 0.8–4 years; RET	Maintained training; subjects were elite athletes	ND in LDH	NA	No reports of muscle tears, greater number of reported muscle cramps in control group	No effect	[135]
20 g/day for 5 days; DB, PC	50 maximal eccentric contractions of elbow flexors	ND in post-exercise CK, LDH between groups	ND in post-exercise strength, ROM between groups	ND in DOMS between groups	No effect	[46]
15.75 g/day for 5 days followed by 5 g/day for 21 mo; OL	Maintained training; subjects were division IA college football players	ND in CK and LDH between groups	NA	NA	No effect	[136]
20 g/day for 5 days; DB, PC	30-km running race	Reduced post-exercise CK, LDH, PGE_2_, TNF-α in creatine group	NA	NA	Beneficial effect	[114]
0.3 g/kg/day for 5 days followed by 0.03 g/kg/day for 5 days; DB, PC	5 sets of 15–20 squats with 50% 1-RM	ND in post-exercise CK, LDH between groups	ND in post-exercise strength, ROM between groups	ND in post-exercise DOMS between groups	No effect	[140]
20 g/day for 5 days; DB, PC	Half-distance Ironman triathlon	Reduced post-exercise TNF-α, IFN-α, IL1-β and PGE_2_ in creatine group, ND in IL-6 between groups	NA	NA	No effect	[110]
0.3 g/kg/day for 5 days followed by 0.1 g/kg/day for 14 days; DB, PC	4 sets of 10 eccentric only leg press, leg extension, and leg curl with 120% concentric 1-RM	Reduced post-exercise CK and LDH (trend *p* = 0.09) in creatine group	Increased recovery of isometric and isokinetic knee extensor strength	NA	Beneficial effect	[112]
0.6 g/kg/day for 5 days; DB, PC	3 sets of 10 of bench press, seated row, leg extension, leg curl, and leg press with 75% of 1-RM	ND in post-exercise CK between groups	NA	NA	No effect	[139]
20 g/day for 7 days followed by 6 g/day for 29 days; DB, PC	7 sets of 10 eccentric leg extension at 150% concentric 1-RM (opposite leg tested at day 30)	ND in post-exercise CK or LDH	ND in post-exercise ROM, dynamic strength, or isometric force at 8 days; increased isometric force at 30 days	ND in post-exercise DOMS	Beneficial effect	[138]
20 g/day for 5 days; DB, PC	Ironman triathlon	Reduced post-exercise CK, LDH, ALD, GOT, GPT, ND in CRP between groups	NA	NA	Beneficial effect	[111]
40 g/day for 5 days followed by 10 g/day for 5 days; DB, PC	6 sets of 10 eccentric contractions at 75°, 90°, and 120° per sec	NA	ND in post-exercise muscle force production	ND in post-exercise DOMS	No effect	[137]
20 g/day for 5 days; DB, PC	Repeated bouts of 4 (14 days apart) sets of biceps curls with 75% of 1-RM to concentric failure	Reduced post-exercise CK in creatine group after second bout	No decrease in post-exercise ROM in creatine group after second bout	Reduced post-exercise DOMS in creatine group after second bout	Beneficial effect	[115]
0.3 g/kg/day for 7 days; DB, PC	Six 35-m maximal sprints with 10-sec rest between bouts	Reduced post-exercise TNF-α, CRP, LDH in creatine group. No effect on oxidative stress markers	NA	NA	Beneficial effect	[113]

*1*-*RM* 1 repetition maximum, *ALD* aldolase, *CK* creatine kinase, *CRP* c-reactive protein, *d* day, *DB* double blind, *DOMS* delayed onset muscle soreness, *GOT* glutamic oxaloacetic acid transaminase, *GPT* glutamic pyruvic acid transaminase, *IFN* interferon, *IL* interleukin, *LDH* lactate dehydrogenase, *mo* month, *NA* not assessed, *ND* no difference, *OL* open label, *PC* placebo controlled, *PGE*_*2*_ prostaglandin E_2_, *RET* retrospective, *ROM* range of motion, *sec* second, *TNF* tumor necrosis, *wk* week

### Creatine Supplementation, High-Force Eccentric Exercise, and Markers of Exercise-Induced Muscle Damage

The effects of creatine supplementation on exercise-induced muscle damage following high-force eccentric exercise have been reported by four groups [46, 112, 137, 138]. Rawson et al. [46] first demonstrated that creatine supplementation (20 g/day for 5 days) had no negative effect on post-exercise serum CK and LDH, strength, range of motion (ROM), swelling, and DOMS. The protocol used by Rawson et al. [46] (50 maximal eccentric contractions) is known to produce marked muscle damage of the elbow flexors [47]. Later, Cooke et al. [112] tested the effects of creatine supplementation (0.3 g/kg/day for 5 days followed by 0.1 g/kg/day for 14 days) on the response to eccentric exercise of the lower body (four sets of ten leg presses, leg extensions, and leg curls with 120% concentric 1 repetition maximum [1-RM]) and found reduced post-exercise CK and LDH (trend) and increased recovery of strength. Using a similar exercise stress (seven sets of ten eccentric leg extensions at 150% concentric 1-RM), Rosene et al. [138] noted that creatine ingestion (20 g/day for 7 days followed by 6 g/day for 23 days) had no effect on CK, LDH, ROM, DOMS, strength, or force on day 8 but improved isometric force production on day 30 after a second exercise bout. Finally, McKinnon et al. [137] reported that creatine supplementation (40 g/day for 5 days followed by 10 g/day for 5 days) ingested for 5 days before and 5 days following a damaging exercise protocol (six sets of ten eccentric contractions of the elbow flexors at 75°, 90°, and 120° per second) had no effect on isometric strength recovery or DOMS. Of note is that even under the most stressful exercise conditions (high-force eccentric exercise), and whether large (knee extensors and flexors) or small (elbow flexor) muscle mass was involved, creatine did not exacerbate the response to this severe muscular challenge. The effects of creatine supplementation on exercise-induced muscle damage following various eccentric exercise challenges are summarized in Table 1 [46, 112, 137, 138].

### Creatine Supplementation, Endurance Exercise, Sprints, and Markers of Exercise-Induced Muscle Damage

Others have examined the effects of creatine supplementation following stressful endurance or sprint exercise (road race, triathlon, repeated sprints) [110, 111, 113, 114]. For instance, Santos et al. [114] reported that 5 days of creatine supplementation (20 g/day) prior to a 30-km race reduced post-exercise CK (19%), prostaglandin-E_2_ (PGE_2_) (61%), tumor necrosis factor (TNF)-α (34%), and completely dampened the increase in LDH. Bassit et al. [110, 111] evaluated the effects of creatine supplementation (20 g/day for 5 days) following half- and full-distance Ironman triathlons. It was noted that the post-race increase in TNF-α, PGE_2_, interferon (IFN)-α, and interleukin (IL)-1-β was blunted following the half-distance Ironman triathlon, as was the post-race rise in CK, LDH, aldolase, glutamic oxaloacetic acid transaminase (GOT), and glutamic pyruvic acid transaminase (GPT) following the full-distance Ironman triathlon in creatine-supplemented athletes. While inflammatory markers are not used clinically in the diagnosis of rhabdomyolysis, they are well-established markers of post-exercise inflammation. Finally, Deminice et al. [113] reported that creatine supplementation (0.3 g/kg/day for 7 days) blunted the post-exercise (six 35-m sprints) increase in c-reactive protein (CRP), TNF-α, and LDH despite increased maximum, mean, and minimum power production. Overall, the available data strongly suggest that creatine supplementation prior to an endurance exercise challenge reduces muscle damage and inflammation in athletes, both of which would predict a lower risk of rhabdomyolysis, not a higher risk. The effects of creatine supplementation on endurance exercise-induced muscle damage are summarized in Table 1 [110, 111, 113, 114].

### Creatine Supplementation, Resistance Exercise, and Markers of Exercise-Induced Muscle Damage

Several groups have investigated the effects of creatine supplementation on recovery from intense resistance exercise that is more typical of real-world training [115, 139, 140]. Rawson et al. [140] examined the effects of creatine supplementation (0.3 g/kg for 5 days; followed by 0.03 g/kg for 5 days) on recovery from a high-intensity squat workout (five sets of 15–20 reps at 50% 1-RM) and found no effect of the creatine on post-exercise strength, serum CK and LDH, ROM, swelling, and DOMS. Machado et al. [139] found no difference in post-exercise CK between creatine- (20 g/day for 5 days) and placebo-supplemented subjects following three sets of ten repetitions of five exercises performed at 75% of 1-RM. Recently, Veggi et al. [115] showed that creatine supplementation (20 g/day for 5 days) attenuated the increase in CK and DOMS and decrease in ROM to a greater extent than a placebo following repeated bouts of resistance exercise (four sets of biceps curls at 75% 1-RM). Together, these papers indicate that creatine supplementation does not disrupt and may enhance recovery from resistance exercise with both concentric and eccentric contractions. The effects of creatine supplementation on resistance exercise-induced muscle damage are summarized in Table 1 [115, 139, 140].

### Creatine Supplementation, Muscle Injury, and Muscle Dysfunction

Several open-label trials of the effects of creatine supplementation on muscle cramps, muscle tightness, muscle strains, injuries (contact and non-contact), missed practices, and players lost for the season in collegiate athletes have been published. In comparison with non-creatine users, creatine users experienced fewer muscle cramps, tightness, strains, and total injuries in 72 collegiate football players, [141] fewer total injuries but no differences in other variables in 39 collegiate baseball players [142], and fewer muscle cramps but no differences in other variables in 130 collegiate football players [143]. In these three studies, creatine ingestion was typical of high-dose loading followed by low-dose maintenance supplementation protocols and lasted from one to three seasons. These data indicate that, in hard-training athletes, creatine may reduce the likelihood of muscle injury and dysfunction. The effects of creatine supplementation on muscle injury and muscle dysfunction are summarized in Table 2 [141–143].

**Table 2:** Effects of creatine supplementation on muscle injury and dysfunction

Supplementation protocol	Population	Muscle cramps	Muscle tightness	Muscle strains	Total injuries	Contact and non-contact injuries	Missed practices and players lost for season	References
0.3 g/kg/day for 5 days followed by 0.03 g/kg/day for 115 days; OL	72 division IA college football players	Fewer in creatine group	Fewer in creatine group	Fewer in creatine group	Fewer in creatine group	ND between creatine and non-creatine users	ND between creatine and non-creatine users	[141]
15.75 g/day for 5 days followed by 5 g/day for rest of season; OL	39 division I college baseball players	ND between creatine and non-creatine users	ND between creatine and non-creatine users	ND between creatine and non-creatine users	Fewer in creatine group	ND between creatine and non-creatine users	ND between creatine and non-creatine users	[142]
15.75 g/day for 5 days followed by 5 g/day for three seasons; OL	130 division IA college football players	Fewer in creatine group	ND between creatine and non-creatine users	ND between creatine and non-creatine users	ND between creatine and non-creatine users	ND between creatine and non-creatine users	ND between creatine and non-creatine users	[143]^a^

*d* day, *ND* no difference, *OL* open label

^a^Only descriptive statistics were presented in this article

### Creatine Supplementation and Case Studies of Rhabdomyolysis

We are aware of six cases of individuals who presented with rhabdomyolysis and were also ingesting creatine [23, 144–146]. While the number of cases relative to the number of individuals ingesting creatine supplements is small, it would be important to identify whether creatine supplementation was an independent factor that precipitated rhabdomyolysis, was a tipping point (perhaps allowing for more contractions/high intensity) in a person already at risk with proven factors (heat, dehydration, NSAID use), or was merely a coincident factor. All of the individuals were adult males (21–33 years) and five of six presented with rhabdomyolysis after participating in extreme unaccustomed exercise (the sixth case was a surgical patient). The involvement of intense exercise indicates exertional rhabdomyolysis rather than creatine supplement-induced rhabdomyolysis. Four of the men were ingesting ephedrine, and both dehydration and herbal diuretic use were also reported, which makes the role of creatine supplements even more difficult to ascertain. Of the six cases, four returned to full function, one returned to partial muscle function, and one died [23]. The fatality was an obese African American man who had recently lost weight (17 lbs in 2 weeks), was dehydrated, was ingesting natural diuretics and ephedrine, and died at the end of a 2-mile run (army fitness test). Given that all of the cases above with a reported link to creatine occurred in young men performing the most common known factor to induce rhabdomyolysis (unaccustomed, heavy exercise) and some were taking other drugs accepted by the FDA as being linked to adverse cardiovascular events (ephedrine), the link with creatine use is highly tenuous, circumstantial, and the likely reason why rational scientific evaluations have not implicated creatine use even in extreme cases of death after excessive exercise and/or dehydration [127].

Despite the severity of these cases (see Table 3), it appears that these are typical cases of exertional rhabdomyolysis. The final case was a patient who presented with rhabdomyolysis following arthroscopic knee surgery [146]. The patient, who was described as a college football player and avid bodybuilder (body mass 100 kg), may have developed rhabdomyolysis as a result of accepted causes of rhabdomyolysis: a recent collision with a tree while skiing (crush syndrome), the tourniquet used during surgery (ischemia), NSAID ingestion (intravenous ketorolac), and pressure on the legs from body positioning during surgery (ischemia). Although not common, cases of tourniquet-induced rhabdomyolysis are mentioned in the literature [147–150], including following arthroscopic knee surgery [146, 151]. Additionally, post-surgery rhabdomyolysis resulting from pressure on the thighs has been reported and pressure-induced rhabdomyolysis in obese patients after bariatric surgery has been reported in patients ingesting ketorolac [152]. It appears that the patient reported by Sheth et al. [146] had numerous factors that could have caused the rhabdomyolysis, including direct muscle injury, ischemia, and medication.

**Table 3:** Case studies of rhabdomyolysis in individuals ingesting creatine

Age and sex	Training status	Creatine supplementation	Clinical manifestations of rhabdomyolysis	Treatment	Potential mitigating factors	Resolution	References
24 M	“Avid bodybuilder”	25 g/day for 1 year	Peak CK > 800,000 IU, severe pain anterior thigh swelling, proteinuria, hematuria, quadriceps compartment syndrome, increased compartment pressure	Emergency tri-compartment fasciotomies, aggressive hydration, physical therapy	8 days no exercise after muscle injury followed by 3 h lower-body resistance training	Quadriceps strength 60% of pre-rhabdomyolysis level at 6 mo post	[144]
33 M	Unknown	Unknown dose/duration use reported by wife	Peak CK 765,910 IU, pain, swelling, myoglobinuria, metabolic acidosis, acute renal insufficiency, increased thigh compartment pressure, thigh compartment syndrome	Hydration; alkalinization, hemodialysis, fasciotomies, resuscitation for cardiac arrest	Post-exercise (collapsed at finish line of 2-mile run, army physical fitness test), obesity, recent rapid weight loss (17 lbs in 2 wk), “natural” diuretics, dehydration, ephedrine, African American	Fatal (multi-system failure)	[23]
27 M	Unknown	5–20 g/day; unknown duration	Peak CK 83,634 IU, myoglobin 30,000 mg/dl	Hydration, alkalinization of urine, hyperbaric oxygen therapy	2 days of “extreme” exercise, ephedrine	Full function	[145]
27 M	Unknown		Peak CK 114,900, myoglobin 20,000 mg/dl, dark urine, oliguria, myalgia, thigh tenderness, increased compartment pressure	Hydration, alkalinization of urine, loop diuretics, continuous venovenous hemofiltration (for respiratory failure), thigh fasciotomy, hyperbaric oxygen therapy	2 days of “extreme” exercise, ephedrine	Full function	
28 M	Unknown		Peak CK > 200,000 IU, myoglobin 23,000 mg/dl, severe pain, dark urine, increased thigh compartment pressures	Hydration, alkalinization of urine, mannitol, loop diuretics, intermittent hemodialysis, thigh fasciotomies	2 days of “extreme” exercise with dehydration, ephedrine	Full function	
21 M	College football player; avid weightlifter	10 g/day for 6 wk	Peak CK 194,000 IU, Peak LDH 15,840 IU, myoglobinuria, severe pain, acute renal failure	IV fluids, IV mannitol, sodium bicarbonate, and furosemide, metoprolol, hydralazine	Hit a tree (crush injury) prior to arthroscopic knee surgery, surgical tourniquet for 100 min, immobilization/positional post-surgical rhabdomyolysis although uncommon, does occur especially in large individuals (pt was 100 kg), hypertension (156/80 mmHg), 15 mg IV ketorolac (NSAID)	Full function	[146]

*CK* creatine kinase, *d* day, *IV* intravenous, *LDH* lactate dehydrogenase, *M* male, *mo* month, *NSAID* non-steroidal anti-inflammatory drug, *pt* patient, *wk* week

## Conclusions

We reviewed the literature on exertional rhabdomyolysis, identified precipitating factors (e.g., unaccustomed exercise [especially with eccentric/muscle-lengthening contractions], ischemia, extreme temperatures, electrolyte abnormalities, endocrinologic conditions, genetic/autoimmune disorders, and drugs), and evaluated the role of statins and creatine supplements in muscle damage and exertional rhabdomyolysis. Healthcare professionals, coaches, and personal trainers must have the knowledge and expertise to recognize when exercise is overly strenuous. Furthermore, they should prescribe exercises and use exercise tests that are safe and will not result in injury. The prevalence of rhabdomyolysis is low in untrained, athlete, and patient populations, but instances still occur where exercise is improperly prescribed or used as punishment, or an incomplete medical history is taken, and exertional rhabdomyolysis can occur. At a minimum, it would be beneficial if all parties involved—athletes and parents, coaches and trainers, and healthcare professionals—were aware of the basic signs of exertional rhabdomyolysis, such as extreme pain and brown urine. Then, rather than a disorganized response and hysterical reaction to what is viewed as a “mysterious disease,” prompt treatment can be administered. Better still, as a preventive measure, all coaches and trainers should be educated on the science of exercise testing and prescription. The design and administration of strength and conditioning programs and exercise fitness testing are supported by many years of research. The leading sport science organizations, such as the American College of Sports Medicine and the National Strength and Conditioning Association have research and applied practitioner journals, position stands, textbooks, professional certifications, and continuing education opportunities to help ensure that the science and practice of exercise testing and prescription is safe and effective.

Regarding creatine supplementation, survey data indicate that creatine monohydrate is a widely used dietary supplement. For example, a survey of Olympic-caliber Finnish athletes indicated that 16% (*n* = 446) used creatine in 2002 and 8% (*n* = 372) used creatine in 2009 [153]. Creatine use in Canadian Olympians was somewhat more common during the Atlanta (14%; *n* = 257) and Sydney Olympic Games (12%; *n* = 300) [154]. A greater prevalence of use was noted in a survey of 133 military (29%) and 96 civilian (57%) health club members [155] and in a survey of 203 Division I collegiate athletes (37%) [156]. The greatest prevalence of use (74%) was reported in 50 Australian power lifters [157].

Although the prevalence of creatine supplementation is quite variable (8–74%), it is clear that many thousands of hard-training individuals are ingesting creatine supplements. If one believed that the link between exertional rhabdomyolysis and creatine supplementation was more than coincident, one would also expect that exertional rhabdomyolysis would occur more frequently in creatine users. There are no such data. As an example, Alpers and Jones [54] reported that the incidence of exertional rhabdomyolysis in military trainees undergoing basic military training was 22 cases per 100,000 per year (0.02%), while Sheppard et al. [155] reported that creatine supplement use in military health clubs was 29%. In fact, given the data that show a protective effect of creatine on muscle inflammation, damage, and injury in hard-training individuals (see Tables 1, 2) and the lack of an obvious increase in exertional rhabdomyolysis in creatine supplement users, it could be argued that creatine supplements are protective against exertional rhabdomyolysis. The 15 studies reviewed in Table 1 demonstrate that creatine supplementation has either no effect on resting, post-resistance exercise, or post-eccentric exercise markers of exercise-induced muscle damage or offers a protective effect during intense endurance or eccentric exercise. The data reviewed in Table 2 demonstrate that intensely training athletes ingesting creatine supplements have similar or reduced muscle dysfunction or injury. It appears that, although often described as a precipitating factor for exertional rhabdomyolysis, there is no evidence to implicate creatine supplementation.
